# Supplementary material for: Veterinary Professionals’ Understanding of Common Feline Behavioural Problems and the Availability of “Cat Friendly” Practices in Ireland
Source: Animals (Basel). 2019 Dec 10;9(12):1112. doi: 10.3390/ani9121112 (PMC6941078; doi:10.3390/ani9121112)
Supplement: Supplementary file 1 [file animals-09-01112-s001.pdf]

Supplementary Material

Online Survey https://www.surveymonkey.com/r/CatBehave

Welcome

Dear Veterinary Professional,

I am a veterinary student at UCD conducting a summer research project in conjunction with Associate Professor Alison Hanlon and Sandra Nicholson MVB. The project explores veterinary professionals' approach to common feline behavioural problems.

We would really appreciate your input to help us gain valuable insight into the current understanding of veterinary professionals in Ireland about treatment options for frequently encountered behavioural problems. This will help support improvements in veterinary education in Ireland and the development of competences in veterinary behavioural medicine.

The survey consists of three sections: Profession role and experience (6 questions), common behaviour scenarios (10 questions), and practice management (5 questions; only relevant to participants in veterinary practice). No identifiable data is requested and so responses are anonymous. The survey closes on *22 July*.

Sincerely,

Matt Goins

15200869@ucdconnect.ie

Consent

1. I give my consent to participate in this project and understand that the data will be used for research purposes and to support the development of veterinary education and competences in veterinary behaviour medicine.
   Agree/Disagree

Section 1: Profession Role and Experience

2. Are you a:
   Veterinary Practitioner (MVB, MRCVS, DVM, etc)
   Veterinary Nurse
   Other (please specify)
3. What year did you graduate?
4. Where was your degree awarded?

   University College Dublin, Ireland

   Dundalk Institute of technology, Ireland

   Other (please specifiy institution and country)

5. How confident do you feel with advising on cat behaviour problems? (Scale: 1-100)
6. Do you currently work in clinical practice?
   Yes
   No *(skip section 3)*

Section 2: Scenarios of Common Feline Behavioural Problems

The following section contains scenarios you may encounter in practice based around common feline behavioural problems. You will be asked to judge how likely each scenario is to result in the best outcome. **The best outcome is defined as one which provides a resolution to the behavioural problem while not compromising the animal's welfare (Shalvey et al, 2019).**

7. While at reception after a check-up, Sally asks the vet nurse, Ciara, why her cat has stopped using the litter box saying, *"We've been having some work done on the house, but he won't even go when the workers aren't there."* Ciara tells Sally, *"Try moving the litter box to a dark, quiet room away from the work and clean up accidents with any ammonia based cleaner. The harsh smell will encourage him to go elsewhere."*
   How likely is this recommendation to give the best outcome?
   Extremely likely /Likely/ Neither likely nor unlikely /Unlikely/ Extremely unlikely/ Don't know
   Comments on your experience of this type of scenario **(optional):**

8. John brings in his four year old unneutered cat, Marmalade, because he's begun spraying next to the back door saying, "*I've also recently noticed the neighbour's cat sitting on the garden wall."*
   The vet advises John to have Marmalade neutered and says, "*You should also make sure to clean the spots well with Dettol, spray the area with Feliway and put something up on the garden wall to block the neighbour's cat."*
   How likely is this recommendation to give the best outcome?
   Extremely likely /Likely/ Neither likely nor unlikely /Unlikely/ Extremely unlikely/ Don't know
   Comments on your experience of this type of scenario **(optional):**

9. Mary brings in her two year old DSH, Penny, for her yearly check-up. She tells the vet, *"Penny won't quit trying to scratch my new couch instead of her scratching posts. What should I do?"* The vet replies, *"Spot test and then spray your couch with Feliway to discourage the scratching. You can also use a catnip spray on the scratching posts to encourage Penny to scratch there instead."*
   How likely is this recommendation to give the best outcome?
   Extremely likely /Likely/ Neither likely nor unlikely /Unlikely/ Extremely unlikely/ Don't know
   Comments on your experience of this type of scenario **(optional):**

10. Anne brings her elderly cat, Bob, in on a repeat visit for a single, large, crusted, non-healing, self-induced ulcer located between the scapulae. Past examinations have ruled out bacterial, fungal, or parasitic infections as well as other common allergens. Due to the unique presentation of the ulcer and having ruled out most purely medical reasons, the vet diagnoses idiopathic ulcerative dermatitis and tells Anne, "*Just keep wrapping the area each time it happens and the ulcer will heal on its own."*
    How likely is this recommendation to give the best outcome?
    Extremely likely /Likely/ Neither likely nor unlikely /Unlikely/ Extremely unlikely/ Don't know
    Comments on your experience of this type of scenario **(optional):**

11. While purchasing flea treatment for her cat, Mary asks the vet nurse, Darren, for advice. *"Whenever my nieces come over, my cat spends the whole day avoiding them and will bolt and then vomit up his dinner. What can I do to reduce his anxiety around them? He never settles."* Darren says, *"Put up some baby gates, blocking off part of your house from your nieces for the cat. Make sure to feed him in one of these rooms."*
    How likely is this recommendation to give the best outcome?
    Extremely likely /Likely/ Neither likely nor unlikely /Unlikely/ Extremely unlikely/ Don't know
    Comments on your experience of this type of scenario **(optional):**

12. During a routine clinical examination, Lorraine asks the vet what she can do to reduce her cat's anxiety during an upcoming move. The vet suggests, "*Get some Feliway diffusers and use them in both houses for at least a few days before the move."*
    How likely is this recommendation to give the best outcome?
    Extremely likely /Likely/ Neither likely nor unlikely /Unlikely/ Extremely unlikely/ Don't know
    Comments on your experience of this type of scenario **(optional):**

13. Coming out of a routine consult, Sara asks the vet nurse "*Socks always gets so scared of the fireworks. With New Year's Eve this weekend, is there anything I can do?"* The vet nurse tells Sara, "*Make sure to stay in so that you can cuddle and reassure him that everything will be okay."*
    How likely is this recommendation to give the best outcome?
    Extremely likely /Likely/ Neither likely nor unlikely /Unlikely/ Extremely unlikely/ Don't know
    Comments on your experience of this type of scenario **(optional):**

14. Clare recently adopted a twelve week old kitten, Tommy. After bringing him in for vaccinations and an exam, she asks the vet for advice because Tommy is nervous around guests. The vet says, *"The best way to solve this is to introduce Tommy to as many different people as possible so that he gets used to it."*
    How likely is this recommendation to give the best outcome?
    Extremely likely /Likely/ Neither likely nor unlikely /Unlikely/ Extremely unlikely/ Don't know
    Comments on your experience of this type of scenario **(optional):**

15. Vanessa has brought her six month old kitten Freckles to the vet for vaccination. She asks how to stop Freckles from attacking her feet. The vet tells Vanessa, *"Get a water gun or spray bottle and spray him whenever he jumps on your feet to discourage him."*
    How likely is this recommendation to give the best outcome?
    Extremely likely /Likely/ Neither likely nor unlikely /Unlikely/ Extremely unlikely/ Don't know
    Comments on your experience of this type of scenario **(optional):**

16. Jo has brought in her two year old cats, Fred and George, for their annual check-up. She asks the vet how to stop Fred from pouncing on and attacking George when he's done using the litter box and says, "*They've used the same litter box since they were kittens. It's only become a problem the last couple of months."* The vet offers her advice, "*You need at least two litter boxes for two cats. Try putting in another one, preferably in an area George frequents."*

How likely is this recommendation to give the best outcome?
Extremely likely /Likely/ Neither likely nor unlikely /Unlikely/ Extremely unlikely/ Don't know
Comments on your experience of this type of scenario **(optional):**

Section 3: Practice Management

17. Does your practice provide any of the following? Tick all that apply:
    - A cat only reception/entrance/waiting area
    - Cat only consultation hours
    - Shelves for cat carriers above "dog level"
    - Towels/covers for cat carriers
    - Television, magazines, or other materials in waiting areas for owners
    - Other (please specify)
18. For clinical consult, does your practice provide any of the following? Tick all that apply:
    - A cat only consult room
    - Use of pheromone products during consults
    - Cat bags or wraps
    - Additional time to allow cats to settle
    - Other (please specify)
19. Which of the following best describes the housing provisions for cat patients in your practice? Tick all that apply:
    - My practice does not have a separate cat ward
    - Cats need to be carried through the dog ward in order to be examined/treated
    - There's machinery located in this room (e.g. washer/dryer machines)
    - There is a set routine for the cat ward (cleaning, feeding, administration of medications, etc at the same time each day)
    - Cats have visual contact with other cats on the ward
    - Dogs may be walked through the cat ward (e.g. to go outside for walks/bathroom)
    - Other (please specify)
20. Regarding the long term hospitalisation of cat patients – which of the following describes the housing and management of these patients? Tick all that apply:
    - Cats are housed in the same cage for the duration of their stay
    - Owners are encouraged to bring bedding, food, and litter from home to match what the cat is accustomed to
    - Other (please specify)
21. Does your practice also board cats?
    - Yes, in the same ward as cat patients
    - Yes, in a separate ward from the dog/cat ward(s)
    - No
    - Other (please specify)

Thank You Message

We'd like to thank you again for taking part in this project. If you'd like to learn more about the research in veterinary behaviour medicine being done at UCD, you can find previous publications at https://doi.org/10.1186/s13620-018-0123-3 and https://doi.org/10.1186/s13620-019-0139-3. You

can also find more information about reducing stress, fear, and anxiety of small animal patients at https://fearfreepets.com.
